# The Benefit of a Retrospective Pregnancy Anamnesis in Child and Adolescent Psychiatry: The Reliability of Maternal Self-Report during Childhood Development

**DOI:** 10.3390/children10050866

**Published:** 2023-05-12

**Authors:** Stefan Mestermann, Peter A. Fasching, Matthias W. Beckmann, Jennifer Gerlach, Oliver Kratz, Gunther H. Moll, Johannes Kornhuber, Anna Eichler, the IMAC-Mind-Consortium

**Affiliations:** 1 Department of Child and Adolescent Mental Health, University Hospital Erlangen, Friedrich-Alexander-Universität Erlangen-Nürnberg (FAU), 91054 Erlangen, Germanyanna.eichler@uk-erlangen.de (A.E.); 2 Department of Obstetrics and Gynaecology, University Hospital Erlangen, Friedrich-Alexander-Universität Erlangen-Nürnberg (FAU), 91054 Erlangen, Germany; 3 Department of Psychiatry and Psychotherapy, University Hospital Erlangen, Friedrich-Alexander-Universität Erlangen-Nürnberg (FAU), 91054 Erlangen, Germany; 4 German Center of Addiction Research in Childhood and Adolescence, University Medical Center Hamburg-Eppendorf, 20246 Hamburg, Germany

**Keywords:** prenatal, pregnancy, self-report, alcohol, smoking, partnership, pregnancy satisfaction, risk perceptions, retrospective

## Abstract

Pregnancy anamnesis is a crucial part of child and adolescent psychiatry diagnostics. In previous works, the reliability of retrospective maternal self-report on perinatal characteristics was heterogeneous. This prospective longitudinal study aimed to evaluate women’s recall of prenatal events in a within-subject design. A sample of 241 women gave a self-report on prenatal alcohol, smoking, partnership quality, pregnancy satisfaction, and obstetric complications during the 3rd trimester (t0), childhood (t1, 6–10 y), and adolescence (t2, 12–14 y). The intra-individual agreement was examined. The t0–t1–(t2) agreement was poor to substantial; this was highest for smoking and worst for obstetric complications, followed by alcohol (Fleiss’ κ = 0.719 to −0.051). There were significant t0–t1–(t2) differences for all pregnancy variables (*p* < 0.017), except for 3rd trimester satisfaction (*p* = 0.256). For alcohol (t0 25.8%, t1 17.4%, t2 41.0%) and smoking (t0 11.9%, t1 16.4%, t2 22.6%), the highest self-reported rates were found during adolescence. During childhood, fewer obstetric complications (t0 84.9%, t1 42.2%) and worse partnerships were reported (t0 M = 8.86, t1 M = 7.89). Thought to be due to social stigmata and memory effects, pregnancy self-reports cannot be precisely reproduced. Creating a respectful and trusting atmosphere is essential for mothers to give honest self-reports that are in the best interest of their children.

## 1. Introduction

Both external and internal stressors influence multiple psychiatric disorders during the prenatal period. Emotional (e.g., anxiety, depression) as well as behavioural disorders (e.g., attention-deficit/hyperactivity disorder [ADHD], conduct disorders, or attachment disorders) are known to be linked to prenatal adversity [1,2,3]. Maternal psychosocial burden, mental illnesses or obstetric complications, notably prenatal toxin exposure, impair child neurodevelopment via fetal programming.

Several studies report the effects of maternal alcohol consumption and smoking during pregnancy on intrauterine development, as these toxins cross the blood-placental barrier [4,5,6]. Worldwide, almost every 10th child is estimated to suffer from prenatal alcohol exposure (PAE), with data peaking in Europe (reaching 25%), despite explicit information and awareness programs as well as high-standard medical care [7]. Severe impairments in a combination of cognitive, sensorimotor, neurophysiological, neurological and psychiatric functioning, as a consequence of maternal drinking, are described as Fetal Alcohol Spectrum Disorder (FASD). Facial stigmata, including microcephaly, hypertelorism, a low nasal bridge, thin upper lip, and smooth philtrum might also be diagnosed in the most severe form (Fetal Alcohol Syndrome, FAS), but are often non-existent in subclinical manifestations [8,9]. Therefore, FASD is often missed and presumably underdiagnosed [10]. A delayed diagnosis limits the therapeutic possibilities and increases the risk of poor clinical outcomes. Hence, early interventions are essential for the best possible quality of life for PAE-affected patients [11]. PAE is also a proven risk factor for ADHD development in childhood and adolescence. However, neurobiological etiology is suspected to differ between PAE-linked ADHD and non-PAE ADHD symptoms [12,13]. 

The global prevalence of smoking during pregnancy is between 14 to 38%, having fortunately decreased since the 1990’s in developed countries. However, the rate of smoking cessation during pregnancy is reported as 27–47%, indicating that between one in four and one in two fetuses of a previously smoking woman are still exposed to tobacco in utero. Furthermore, the literature indicates an increasing prevalence in developing countries [14]. Smoking and stand-alone nicotine consumption (e.g., via e-cigarettes) may induce embryotic physical abnormalities such as reduced head size or body length, limb reduction, and orofacial clefts. Further effects are cardiovascular risk profiles, obstructive respiratory diseases, impaired immune system responses, and an increased risk of the fetus being preterm or stillborn [15,16]. Besides somatic effects, in utero nicotine and smoking exposure is likely to cause neurocognitive (executive or sensory) functioning and behavioural problems such as ADHD or conduct disorders. The risk of nicotine addiction during the offspring’s adulthood is also increased [17]. The impetus of maternal smoking on other mental diseases such as anxiety or depression is discussed heterogeneously [14,18]. Nevertheless, a history of toxin contact of any kind is hence more likely among psychiatric patients.

Obstetric complications, e.g., bleeding, nausea, gestational diabetes mellitus, preeclampsia or infections, not only impact the infant’s somatic outcome, but also take effect indirectly. Prenatal psychosocial factors such as mothers’ stress level, mood, and partnership are proven to influence the offspring’s psychological and neurological development, cognitive ability, and fearfulness [19,20]. As mechanisms, altered cortisol release and glucocorticoid receptor sensitivity via DNA-methylation as well as changes in neuropeptides, neurotransmitters (e.g., glutamate), and impairment of the hypothalamic-pituitary-adrenal-axis have been identified [21,22,23]. Furthermore, epigenetic variations of the oxytocin receptor genotype are discussed to influence the infant’s susceptibility to stress [24]. Changes in the cortical neural structures responsible for cognitive-emotional, sensory, and socio-emotional functioning were identified via diffusion-weighted MRI scans [25]. The literature indicates an increased risk for mental illnesses in the offspring, such as anxiety, depression, and externalising disorders, as consequences of maternal psychosocial stress during pregnancy [26].

Based on the increasing prevalence of various psychiatric disorders among minors and growing parental stress levels, not least because of the COVID-19 pandemic, sufficient and impactful diagnostics in child and adolescent psychiatry are essential to identify pathological mechanisms and provide effective therapy solutions [27,28]. Current medical practice guidelines demand a sufficient pregnancy and birth anamnesis, particularly with parents or further caregivers, or both, when assessing mental disorders among minors [29,30,31]. The International Classification of Diseases (ICD-10) describes disorders originating in the perinatal period in chapters P90–96 [32]. The Diagnostic Classification of Mental Health and Developmental Disorders of Infancy and Early Childhood (DC: 0–5) includes ‘prenatal conditions and exposures’ (e.g., to medications, alcohol, other substances or teratogens) on axis III (physical health conditions and considerations) and ‘pregnancy-related stressors’ to be diagnosed on axis IV (psychosocial stressors) [33]. ICD-11 redefines these categories under Chapter 19: ‘certain conditions originating in the perinatal period’, specified as ‘Fetus or newborn affected by maternal factors or by complications of pregnancy, labour or delivery’ and exposure to ‘noxious influences transmitted via placenta or breast milk’ [34]. 

Thus, for child and adolescent psychiatry clinicians, pregnancy anamnesis is a crucial tool for diagnosis. Exposure-specific biomarkers for detecting maternal prenatal substance use are current subjects of research but are still limited in their validity for clinical usage. Furthermore, these biomarkers are limited to prenatal utilization and are not available during later child and adolescent psychiatric assessments [14,35]. Therefore, third-party interviews (e.g., with parents) most commonly in face-to-face or questionnaire format, or both, are a viable and easy-to-perform diagnostic method. Pre- and perinatal stress is often assessed by various instruments, for instance using the ‘Prenatal Distress Questionnaire’, ‘Pregnancy Experience Scale’ or ‘Prenatal Social Environment Inventory’ [36,37]. They are expected to provide substantial information on patients’ symptom development and possible psychosocial and biological influencing factors. However, this method has several limitations, pointed out by mothers differing in (repeated) interviews about their gravidity in previous works. Suspected reasons for under- or over-reporting include recall biases, e.g., because of long time gaps or maternal psychiatric disorders that may influence memory and rumination biases. Furthermore, the concealment of substance use or maternal psychological stress is possible due to social stigmata, social desirability or conscience issues, where cultural and ethnic influences may play a role as well [38,39,40,41]. Hence, the potential development of adolescent psychiatric disorders in (initially) subclinical children is a considerable risk.

Study aims: The aim of this longitudinal prospective cohort study, with three points of data collection, was to validate women’s subjective self-reports regarding hard-risk pregnancy factors, such as substance use (alcohol, smoking). In addition, self-reports of emotional soft-risk pregnancy characteristics, such as psychosocial parameters, were evaluated. To date, their assessment is not generally standardized, and therefore, pregnancy risks are difficult to detect, resulting in a low number of studies addressing this concern. Current literature addresses maternal recall validity predominantly via single- or two-point data collection and comparison to mostly objective birth data collection in the perinatal examination, postnatal visits or national databases [19,38]. We intended to review the gap in evaluating subjective parental interviews as an integral element of anamnesis. We also aimed to detect potential changes in mothers’ self-report during their children’s development and discuss possible age-specific contributing factors.

## 2. Materials and Methods

### 2.1. Study Design and Sample Characteristics

**Figure 1:**
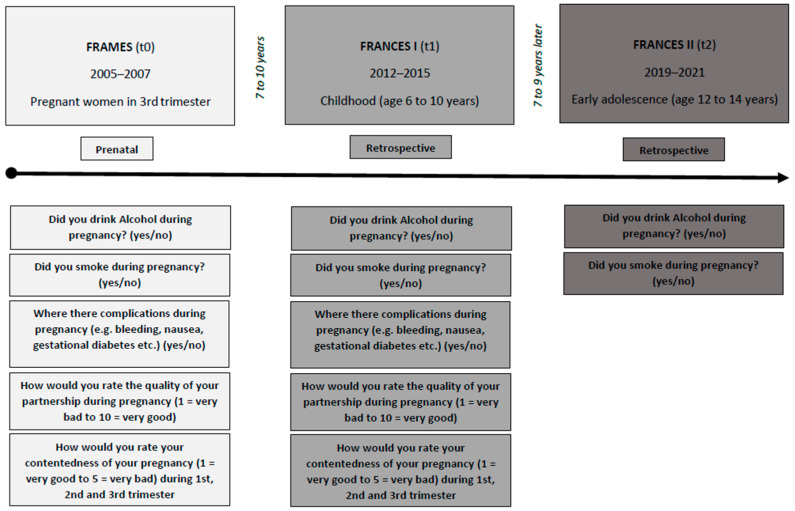
The study design and questioning of FRAMES (t0), FRANCES I (t1) and FRANCES II (t2).

This study is based on data from the Franconian Cognition and Emotion Studies (FRANCES) [12,42], a follow-up cohort study of the prospective longitudinal Franconian Maternal Health Evaluation Studies (FRAMES) [43]. Women in their 3rd trimester, with a minimum age of 18 years and 30 weeks of gestation time, were recruited at the Department of Obstetrics and Gynecology (n = 1100) from 2005 to 2007 (t0). The 2nd (t1) and 3rd (t2) assessments were conducted at the Department of Child and Adolescent Mental Health. Between 2012 and 2015, when children attended primary school (age 6 to 10 years), a subsample with n = 618 (56.2%) of these women was contacted again for re-participation in the FRANCES I follow-up (t1). This FRANCES I cohort was contacted again from 2019 to 2021, during the children’s early adolescence (age 12 to 14 years), for a second data acquisition (FRANCES II, t2) [9]. Out of n = 618 contacted FRAMES-women, n = 245 (with n = 248 children) participated at t1 (39.6%; age of children: M = 7.74, SD = 0.74, range 6.00–9.90). All families who attended t1 were contacted again; 76% (n = 186 mothers with n = 188 adolescents) of the FRANCES I sample re-participated in FRANCES II (age of children: M = 13.3, SD = 0.34, range 12.8–14.5) (see Figure 1). When comparing t2 participating families with t2 non-participating families, no differences in marital status (χ^2^(1) = 0.35, *p* = 0.552), family income (χ^2^(4) = 3.94, *p* = 0.414) or maternal total psychopathology (t(234) = −0.93, *p* = 0.353) at t1 were found. However, higher-educated mothers were more willing to re-participate (χ^2^(1) = 7.60, *p* = 0.006). 

For present data analyses, after the exclusion of two twin pairs, valid t1 data of n = 241 and valid t2 data of n = 183 mother-child dyads were available. It was first gravidity for 99 women (41.1%) of the cohort, second for 75 (31.1%) and third or higher for 77 (27.8%). A total of 190 mothers (78.8%) were nullipara. A total of 228 mothers (94.6%) had German nationality, and 13 (5.4%) had another (including dual) citizenship. At t0, 73 mothers (30.8%) rated above the cut-off for possible depression on the Edinburgh Postnatal Depression Scale (EPDS) [44], and the cohort’s mean t0-rating was below the cut-off (M = 6.86, SD = 4.90). In the follow-ups, 59 (25.4%) mothers at t1 and 20 (10.9%) at t2 self-reported depression. The demographic sample characteristics are summarized in Table 1 and the study design is shown in Figure 1.

### 2.2. Prenatal and Retrospective Self-Report

Alcohol consumption (t0, t1, t2): At t0, women were asked in a structured interview (face-to-face format) about their drinking and smoking behaviour during pregnancy. The question ‘Did you drink alcohol during your current pregnancy?’ could be answered with the response alternatives: ‘No, I generally do not drink’, ‘No, I did not drink during pregnancy’, ‘Yes, I rarely drank during pregnancy’, ‘Yes, I drank 1 glass /day during pregnancy’ and ‘Yes, I drank more than 1 glass /day during pregnancy’. For t1 and t2, women answered standardized questions (paper-and-pencil format) with the options: ‘No, I did not drink alcohol during my pregnancy’ or ‘Yes, I did drink alcohol during my pregnancy’, followed by a quantification via glasses per day/week/month/trimester. For analysis, data were dichotomized: Women declaring no alcohol consumption during pregnancy (0 = ‘I did not drink alcohol during pregnancy’) vs. women declaring alcohol consumption during pregnancy (1 = ‘I drank alcohol during pregnancy’). 

Smoking behaviour (t0, t1, t2): In the 3rd-trimester interview and t1/t2 questionnaires, women chose between the following options: ‘No, I am a non-smoker’, ‘Yes, I smoked before pregnancy’, ‘I have stopped smoking since I knew I was pregnant’ and ‘Yes, I smoke(d) during pregnancy’. Where applicable, participants were asked to quantify the number of cigarettes smoked per day. The data were also split into a nominal scale: Women stating no nicotine consumption (0 = ‘No, I am a non-smoker’ and ‘Yes, I smoked before pregnancy’) versus women stating nicotine consumption (1 = ‘Yes, I smoked during pregnancy’, ‘I have stopped smoking since I knew I was pregnant’).

Obstetric complications (t0, t1): Mothers provided information on the dichotomous categories: ‘There were complications during my pregnancy’ or ‘There were no complications during my pregnancy’, including further differentiation where necessary (e.g., bleeding, nausea, gestational diabetes, among others).

Partnership quality and pregnancy satisfaction (t0, t1): For an estimation of prenatal partnership quality and pregnancy satisfaction during the 1st, 2nd, and 3rd trimesters, Likert-Scale ratings were given by the mothers (partnership: 1 = very bad to 10 = excellent; pregnancy satisfaction analogous to German school grades 1 = very good to 5 = very bad).

### 2.3. Statistical Analyses

Data were analyzed using IBM^®^ SPSS^®^ Statistics (Version 24.0). The level of analysis significance was defined as *p* < 0.05 (two-tailed). Normal (Gaussian) distribution was evaluated via the Kolmogorov-Smirnov test. In the first step, intraindividual agreement was determined by Fleiss’ (3 points of data collection) or Cohen’s (2 points of data collection) Kappa (κ) and interpreted as <0.00 (poor), 0.00–0.20 (slight), 0.21–0.40 (fair), 0.40–0.60 (moderate), 0.61–0.80 (substantial), and 0.81–1.00 (almost perfect) for categorical data. For interval-scaled continuous variables, the intra-individual agreement was calculated using Spearman’s correlations (rs), with |rs| ≥ 0.10 considered low, |rs| ≥ 0.30 moderate, and |rs| ≥ 0.50 strong or high relations.

Additionally, group comparisons for repeated or dependent measures were performed by Cochran’s Q Test with post hoc Dunn Test (alcohol consumption and smoking behavior: dichotomous at t0, t1, t2), McNemar Test (obstetric complications: dichotomous at t0, t1), Wilcoxon signed-rank test with effect size measure r, interpreted as 0.1–0.3 (weak), 0.3–0.5 (moderate) and >0.5 (strong) (partnership quality and pregnancy satisfaction: continuous at t0, t1). An overview of the data scale levels and the corresponding statistical tests are shown in Table 2.

## 3. Results

### 3.1. Alcohol Consumption

**Figure 2:**
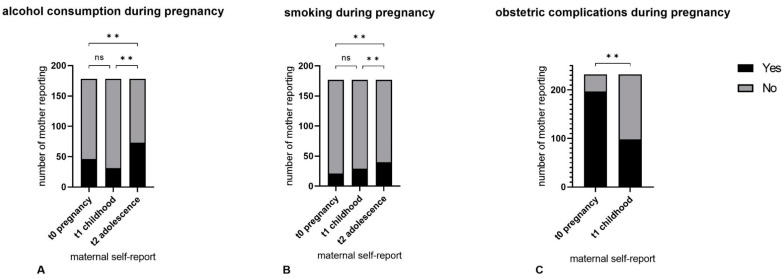
The t0–t1–t2 self-report data on alcohol (**A**), smoking (**B**) and obstetric complications (**C**) during pregnancy. ** *p* < 0.01, ns: not significant.

Figure 2A shows the t0–t1–t2 self-report data. Fleiss’ κ was slight (κ = 0.203) for mothers’ t0–t1–t2 reports on alcohol consumption. Cochran’s Q showed significant differences for positive t0–t1–t2 answers (‘Yes, I did drink alcohol during my pregnancy’) (*p* < 0.001), for which the post hoc analysis yielded a significantly higher number of positive recalls at t2 (n = 73; [41.0%]) compared to t1 (n = 31; [17.4%]; *p* < 0.01) and t0 (n = 46 [25.8%]; *p* = 0.001). No significant differences were shown between t0 and t1 (*p* = 0.143) (see Table 3).

### 3.2. Smoking Behaviour

Figure 2B shows the t0–t1–t2 self-report data. Maternal self-reports on prenatal smoking behaviour yielded substantial t0–t1–t2 agreement (Fleiss’ κ = 0.719; *p* < 0.01). Cochran’s Q showed significant t0–t1–t2 differences (*p* < 0.01). Post hoc analysis revealed a significantly higher number of positive recalls at t2 (n = 40; [22.6%]) compared to t1 (n = 29; [16.4%]; *p* = 0.01) and t0 (n = 21; [11.9%]; *p* < 0.001). No significant differences were found between t0 and t1 (*p* = 0.98) (see Table 3).

### 3.3. Complications during Pregnancy

Cohen’s κ showed poor strength of t0–t1 agreement (κ = −0.051). Significantly fewer mothers reported pregnancy or birth complications at t1 (n = 98 [42.2%]) compared to t0 (n = 197; [84.9%]; *p* < 0.001) (see Figure 2, Table 3).

**Figure 3:**
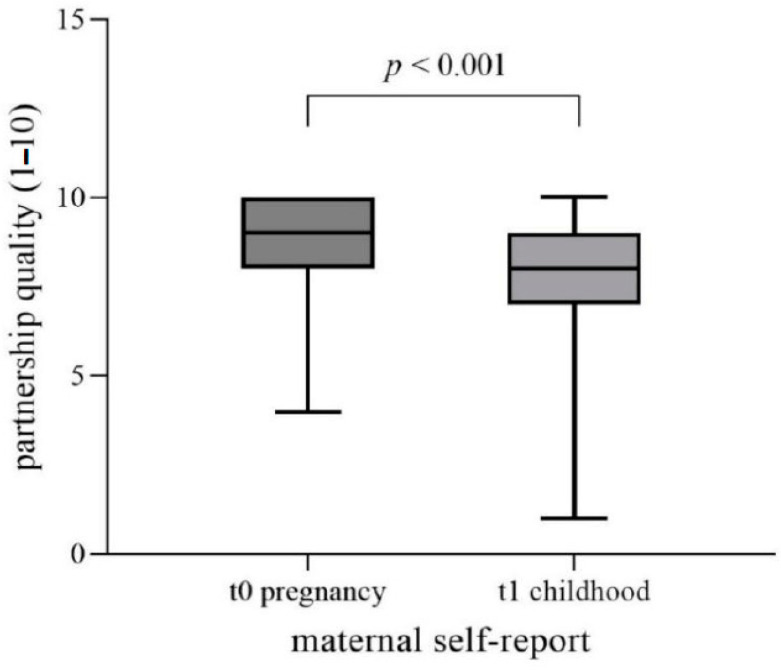
The t0–t1 self-report data for partnership quality during pregnancy. Rating of partnership quality via Likert-Scale: 1 = very bad to 10 = excellent.

Figure 3 shows the t0–t1 self-report data. The t0–t1 agreement of mother-rated partnership quality during pregnancy was moderate (Spearman’s t0–t1 correlation r_s_ = 0.371, p_r_ < 0.01). Wilcoxon signed-rank test yielded a significantly lower estimation of partnership quality at t1 (M = 7.89 ± 2.05) than at t0 (M = 8.86, SD = 1.19; *p* < 0.001) at a nearly strong effect size (*r* = 0.49) (see Table 4).

### 3.4. Subjective Satisfaction with Pregnancy

**Figure 4:**
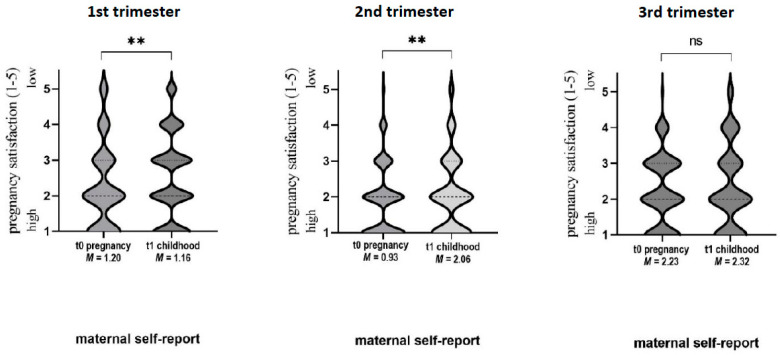
The t0–t1 self-report data for pregnancy satisfaction. Rating of pregnancy satisfaction via Linkert-Scale analogous to German school grades 1 = very good to 5 = very bad: ** *p* < 0.01, ns: not significant, *M*: mean.

Figure 4 shows the t0–t1 self-report data. For all trimesters, the t0–t1 agreement of mother-rated pregnancy satisfaction was moderate (Spearman’s t0–t1 correlation for 1st trimester: r_s_ = 0.544; p_r_ < 0.01; 2nd trimester: r_s_ = 0.525; p_r_ < 0.01; 3rd trimester: r_s_ = 0.467; *p* < 0.01). Wilcoxon signed-rank test yielded significant differences in pregnancy satisfaction reports for 1st and 2nd trimesters (however, not for 3rd trimester, *p* = 0.256): for the 1st trimester, subjective pregnancy satisfaction was reported higher (resulting in lower values) at t1 (M = 2.28, SD = 1.16) compared to t0 (M = 2.50, SD = 1.20) (*p* = 0.003); for the 2nd trimester, vice versa (t1: M = 2.06 ± 1.03; t0: M = 1.91 *±* 0.93; *p* = 0.017) (see Table 4).

## 4. Discussion

This study examined the retrospective reliability of women’s self-report on pregnancy events in a longitudinal within-subject design study. We intended to detect potential changes in the mothers’ self-reports from pregnancy over childhood and into adolescence to give clinicians a valid basis for self-report interpretation in child and adolescent mental health anamnesis. For that purpose, we tried to differentiate more reliable from less reliable domains of maternal retrospective pregnancy anamnesis. We compared current and retrospective maternal self-reports on prenatal substance use, obstetric complications, and psychosocial well-being at three measurement points (pregnancy, childhood, and adolescence).

Substance use: The maternal self-report on prenatal alcohol consumption fluctuated. We demonstrated an over-time decrease (childhood) followed by a nearly doubled increase (adolescence), with the highest rates in adolescence. The intra-individual agreement was only slight. This corresponds with earlier findings, where retrospective maternal ratings of alcohol use showed low agreement with birth data: Ramos et al. reported κ = 0.23 within seven and a half years [45]. Hannigan et al. showed significantly higher reports of drinking during adolescence (age 14 y) than at birth, with an over tenfold increase (*p* < 0.001) in consumed alcohol, matching with, and even surpassing, our results [46]. Similar findings were demonstrated by Jacobson et al. in 2002, where mothers reported higher alcohol consumption at t1 (M = 0.88 oz/d) than before birth (M = 0.23 oz/d; *p* < 0.001) but with a higher agreement (r = 0.60, *p* < 0.001) than our data. It is worth noting that they applied a shorter t0–t1 gap (13 months after birth) [41]. The TRAILS Study revealed a low Cohen’s κ for recollection agreement (κ = 0.03–0.11), similar to our results, and concluded over-reporting [38] as here the national birth databank was taken as the gold standard (‘true’ data), and so under-reporting at birth was assumed. Even further studies support the under-reporting of alcohol consumption during pregnancy [47,48]. If the probability of obtaining reliable self-reports on prenatal alcohol consumption increases with child age, our data suggest a substance use rate of >40% in a German general population cohort.

The literature on prenatal smoking, however, as demonstrated by our data, shows more consistent maternal self-reporting. We were able to replicate substantial retrospective agreements in this regard, published by TRAILS (κ = 0.77, with balanced rates of over- and under-reporting around 5–6%) [38], Pickett et al. (κ = 0.75) [39] and Ramos et al. (κ = 0.65) [45]. It has to be noted that these studies used a longitudinal study design with only two points of data collection, whereas our study design included three (t0–t1–t2). Furthermore, our statistical tests confirm the agreement between t0 and t1 (6 to 9 years after pregnancy report) regarding smoking during pregnancy but indicate significant differences compared to questioning after a longer period (12 to 14 years after pregnancy report, t2). Similar to alcohol consumption, maternal report rates raised from pregnancy to adolescence, as our data revealed a doubling of positive smoking reports from offspring’s birth to adolescence. This is supported by Czeizel et al., who postulated low reliability for retrospective self-reports on prenatal smoking in a cohort of children with suspected nicotine-related congenital defects [48]. Similar to the self-reports on prenatal drinking, women might under-report tobacco use at birth or during childhood and primarily come out with ‘true’ statements later on. According to cultural norms [38,39,40,41], cognitive avoidance behaviour—during pregnancy due to social stigmata, during childhood due to fear of negative consequences for child development—might contribute to the mother’s negation of prenatal alcohol consumption [42]. Recall biases certainly play a role but cannot explain the systematically higher reported rates in adolescence. More precise retrospective self-reports on prenatal smoking behaviour might be explained by less social stigma or less public knowledge of child developmental consequences, or both. 

For the clinical setting, our results confirm the highest reliability of maternal retrospective anamnesis on prenatal substance use during the offspring’s adolescence. At this age, youths often present in the clinical psychiatric setting with symptoms of depression, anxiety, eating disorders or substance use. At primary school age, patients predominantly show symptoms of ADHD, conduct disorders, emotional disorders or autism spectrum disorders [49]. Particularly in ADHD and conduct disorders, the literature stated the predisposing risk of prenatal alcohol and smoking exposure [12,13,14,18]. Thus, clinicians are troubled because the maternal retrospective self-report on substance use during pregnancy seems less reliable during this illness-specific age period. Impaired diagnostic procedures might result in delayed diagnosis and therapy. Diagnostic awareness and sufficient methods are required to gather this information, particularly during pathognomonic periods. Third-party interviews, e.g., fathers or relatives, are further diagnostic sources. In our study, no data from third-party evaluations were included, which also poses a limitation. First and foremost, creating a respectful and trusting atmosphere is essential to enable the mother herself to give reliable self-reports, free of shame and in the best interest of the child. 

Obstetric complications: Our data showed a significantly smaller number of obstetric complications reported at t1 compared to the pre-birth assessment. The number of reported complications halved from pregnancy to childhood. This reinforces the findings by Dietz et al., who reported poor retrospective maternal agreement, particularly on placenta praevia and urinary tract infections [50]. They hypothesize that women missing medical knowledge, unspecific questioning, and recall bias as possible explanations. Ramos et al. demonstrated a heterogeneous retrospective agreement for obstetric complications self-reports, strongly depending on differentiated types of medical problems [45]. As our questioning was widely generalized, obstetric complications were less likely to be retrospectively assessed. Recall bias remains a notable limitation in our study as in earlier works.

Psychosocial prenatal risks: Partnership quality and personal satisfaction during pregnancy are social and psychological dimensions of maternal stress [19]. Partner support is suggested to have an important influence on maternal satisfaction, therefore influencing fetal outcomes and child health [51,52]. Multiple factors influence the rating of these complex domains, making standardized assessment difficult. Our data indicate a higher estimation of partnership quality during pregnancy compared to the offspring’s childhood. Is the first or the second rating the gold standard in this context? During pregnancy, the joyful anticipation of having a baby might strengthen the feeling of togetherness. Later, when children grow up, parenting could be seen as more differentiated. Additionally, postnatal partnership conflicts might result in biased retrospective ratings. In clinical child and adolescent psychiatric anamnesis, it thus proves crucial to retrospectively consider mothers’ partnership and support as protection or risk factors during pregnancy, as stated in the literature [2].

Quality of life and satisfaction during pregnancy have become relevant parameters in clinical treatment, however, measurements are not widely standardized [53,54]. Analysis of our data on subjective pregnancy satisfaction showed heterogeneous trimester-depending results. Retrospectively, there were upgrading tendencies for the 1st trimester and downgrading tendencies for the 2nd trimester. For the 3rd trimester, the agreement was good and without significant differences. One explanation for the trimester-depending results could be that bio-psycho-social dimensions of stress influence general satisfaction differently at specific periods. Most typical 1st-trimester complications are nausea and vomiting [55]. Especially a general, unspecific malaise during the 1st trimester seems difficult to remember in retrospect. In contrast, subjective pregnancy satisfaction during the 3rd trimester might be easier for mothers to reproduce. For clinicians addressing child and adolescent mental health, our data show that the retrospective ratings of maternal satisfaction during the 3rd trimester of pregnancy were the most reliable to work with.

## 5. Conclusions

Due to social stigmata and memory effects, subjective pregnancy self-reports cannot be precisely reproduced in retrospection. Nevertheless, there is an intra-individual relevant pregnancy-childhood (-adolescence) correlation for all presented pregnancy risks. That is why the maternal retrospective information is not random and is valuable for anamnesis in child and adolescent psychiatry. However, the child and adolescent health specialist should keep in mind that especially pregnancy complications are retrospectively under-reported, fluctuating substance use is reported, with increasing—and perhaps more realistic—amounts from childhood to adolescence, the partnership could be devalued in retrospect, with 3rd-trimester satisfaction being the most reliable marker. Clinicians should be aware of potentially biased maternal information, particularly regarding prenatal substance use in the treatment of children at elementary school age. Despite all inconsistencies, the mother is the most important source of information for intrauterine stress in child and adolescent psychiatric anamnesis. Other child caregivers—who have already been present during pregnancy—can be additionally consulted. Apart from details, creating a respectful and trusting atmosphere is essential to enable the mother to give reliable self-reports, free of shame and in the best interest of the child.

## Figures and Tables

**Table 1:** The sample characteristics (mothers, fathers, children) and over-time differences.

		FRAMES t0	FRANCES I t1	FRANCES II t2
		M (SD)/N (%)		
Mothers				
Age [y]		32.6 (4.7)	40.4 (4.4)	46.0 (4.4)
BMI (prepartum) $\left[\frac{kg}{m^{2}}\right]$		24.1 (5.5)		
Marital status	Single Married	194 (80.5) 47 (19.5)	203 (85.7) 34 (14.3)	151 (82.7) 32 (17.5)
Years of school attendance	>12 ≤12	127 (52.7) 114 (47.3)	129 (53.5) 112 (46.5)	112 (61.2) 71 (38.8)
Family income (monthly, net)	<1000 € 1000–2000 € 2000–3000 € 3000–4000 € 5000–4000 € >5000 €	5 (2.8) 35 (19.6) 61 (34.1) 37 20.7) 23 (12.8) 18 (10.1)	0 (0.0) 23 (9.5) 54 (22.4) 50 20.7) 48 (19.9) 66 (27.4)	0 (0.0) 9 (4.9) 23 (12.6) 38 (20.8) 37 (20.2) 76 (41.5)
Fathers				
Age [y]		35.4 (5.5)	43.2 (5.5)	48.7 (5.5)
Children				
Age [y]			7.7 (0.76)	13.3 (0.34)
Sex	Female Male	117 (48.5) 124 (51.5)	117 (48.5) 124 (51.5)	90 (49.2) 93 (50.8)

Notes: M: mean value, SD: standard deviation, N: Number of participants, F/T: test statistics, df: degrees of freedom, η2/d/κ: tests’ effect size.

**Table 2:** The data arrangement and statistic methods.

Maternal Self-Report on Prenatal…	FRAMES (t0)	FRANCES I (t1)	FRANCES II (t2)	Scale Level	Intraindividual Agreement	Dependent Group Comparison
Alcohol	+	+	+	Nominal	Fleiss’ κ	Cochran’s Q
Smoking	+	+	+	Nominal	Fleiss’ κ	Cochran’s Q
Complications	+	+	−	Nominal	Cohen’s κ	McNemar
Partnership	+	+	−	Interval	Spearman’s r	Wilcoxon
Satisfaction	+	+	−	Interval	Spearman’s r	Wilcoxon

**Table 3:** The statistics for maternal alcohol consumption, smoking, and complications during pregnancy (dichotomous items).

Maternal Self-Report on Prenatal…		3rd Trimester t0 (n = 241) FRAMES	Primary-School t1 (n = 241) FRANCES I	Adolescence t2 (n = 183) FRANCES II	Statistics				
					*p*	Post-Hoc *p*			κ
	N (%)					t_0_/t_1_	t_1_/t_2_	t_0_/t_2_	
Alcohol	Yes No	46 (25.8) 132 (74.2)	31 (17.4) 147 (82.6)	73 (41.0) 105 (59.0)	<0.001	0.143	<0.01	0.001	0.203 **
Smoking	Yes No	21 (11.9) 156 (88.1)	29 (16.4) 148 (83.6)	40 (22.6) 137 (77.4)	<0.001	0.98	0.01	<0.001	0.719 **
Complications	Yes No	197 (84.9) 35 (15.1)	98 (42.2) 134 (57.8)	- -	<0.001	-	-	-	−0.051

Notes: N: Number of participants. *p:* Cochran’s/NcNemar’s level of significance, post hoc *p*: level of significance of post hoc Dunn test, ** *p* < 0.01; κ: Cohen’s or Fleiss’ kappa.

**Table 4:** The statistics for mother ratings of quality of partnership and subjective satisfaction with pregnancy (continuous items).

Maternal Self-Report on Prenatal…	3rd Trimester t0 FRAMES			Primary-School t1 FRANCES I					
	M	SD	Range	M	SD	Range	*p*	*r*	r_s_
Partnership (1–10)	8.86	1.19	6	7.89	2.05	9	<0.001	0.49	0.371 **
Satisfaction (1–5)									
1st tri. 2nd tri. 3rd tri.	2.50 1.91 2.23	1.20 0.93 0.99	4 4 4	2.28 2.06 2.32	1.16 1.03 1.12	4 4 4	0.003 0.017 0.256	0.20 0.16 0.08	0.544 ** 0.525 ** 0.467 **

Notes: M: mean value, SD: standard deviation, T: range, *r:* effect size (of Wilcoxon signed-rank test), r_s_: correlation coefficient (Spearman), tri = trimester, sample size n = 241, ** *p* < 0.01. Partnership quality during pregnancy. Pregnancy satisfaction.

## Data Availability

The datasets generated during and/or analysed during the current study are available from the corresponding author on reasonable request.
